# The Therapeutic Value of Cephalic and Spinal Electrizations*This paper was read before the Association of Superintendents of American Institutions for the Insane, at Toronto, Canada, June 14th, 1881, some of the clinical records being then briefly detailed verbally.

**Published:** 1883-03

**Authors:** G. H. Hughes

**Affiliations:** St. Louis


### THE THERAPEUTIC VALUE OF CEPHALIC AND SPINAL ELECTRIZATIONS.* BY G. H. HUGHES, M.D., ST. LOUIS.

The physiological experiments of MM. Latournian and Laborde (*Gazette Hebdominaire*, 1879), confirmed by those of MM. Condorceau and Duval, performed on inferior animals, have fully demonstrated the power of electrizations to produce in the brain a state of temporary anæmia immediately following each application. But these demonstrations were only confirmations of a fact previously ascertained by clinical methods. M. Latournian having himself before reported the case of the Abbé C., whose brain, chronically congested to such a degree as to produce marked and grave psychical aberrations, yielded favorably to persistently repeated cephalic electrizations, and I had myself, long before this, employed these applications for this purpose, and became convinced, from repeated experience, of their power over the brain to tranquilize and subdue cerebral excitation, and over the vasomotor system to promote through them the contraction to normal dimensions of the abnormally distended cerebral arterioles in hyperæmic encephalic conditions.

Though the precise mode of action of the constant current in destructive brain lesions will probably not be understood until a number of cases, which have been treated in a similar manner, have been investigated post mortem, as Althaus says, is perhaps true; yet we now understand its modus operandi in cerebral congestion quite well, and in this knowledge we have, in part, doubtless, a comprehension of how it may act in arresting, if not in diminishing, the growth of morbid products within the brain. The diminished caliber of the cerebral vessels may be inimical to their development, and the same influence that restores normal vasomotor tonicity, may extend itself to the trophic and absorbent systems.

In the beginning of 1878 it had become quite a routine practice with me to so employ the constant galvanic current, and I have the records of a number of cases of induced cerebral hyperæmia,

* This paper was read before the Association of Superintendents of American Institutions for the Insane, at Toronto, Canada, June 14th, 1881, some of the clinical records being then briefly detailed verbally.

one of them a case of *meningitis verticalli*, which occurred in a late general of the army, as the results of a saber wound received in battle, in which the effects of repeated applications of this valuable therapeutic agent were most salutary. Since then I have extended the employment of the constant current to all well-marked congestive states of the cerebro-spinal nervous system, and to parts so involved, and intimately associated with the sympathetic system.

We may say, before passing to the record of our cases, that a now somewhat extended observation in electro- and neuro-therapy seems to confirm what Löwenfeld deduced from experimental galvanization of rabbits, viz: that, while descending currents contract (the vessels of the encephalon including its) meninges, ascending currents, from neck to forehead, dilate them; and it is well, also, to bear in mind Löwenfeld's further assertion that cross currents dilate on the side of the anode and contract on that of the cathode, while induced currents in any direction cause *hyperœmia cerebri*. These facts may also be satisfactorily proven by personal experimentation, and the failure to appreciate them is at the foundation of the ill success of so many who have attempted to employ cephalic electrization for therapeutic purposes and discarded it. It has not, in these instances, been the electric current which has been at fault, but the operator who has misdirected it. It is as valuable a servant, when skillfully used, as the surgeon's knife, and we should not condemn it because, in unskillful hands, it may prove equally unsafe aund unsatistactory.

In the present note we content ourselves with a few clinical confirmations of the value of the constant descending current in conditions of the brain associated, primarily or secondarily, with hyperæmia, reserving for another time illustrations of its value in other cerebral states and in certain abnormal conditions of the spinal cord.

Althaus, *vide* "*Brain*," April, 1881, has employed this agent successfully in resolving morbid depositions within the brain, and we have seen hemiplegia, dysphagia and aphasia from lesions of the brain and pons, disappear under its use, and the conviction has forced itself upon us from the more satisfactory results since its regular employment in our treatment of our epilepsias, conjoined with internal therapy, that it is an auxilliary in this affection which ought not to be despised. True, these cases recover under treatment without galvanism, but if the majority of our cases under the combined treatment stay well, whereas formerly the most of them, perhaps three-fifths, relapsed, it is not unreasonable to have acquired a little faith in its aid.

Althaus (*vide supra*) has successfully treated diabetes insipidus by galvanizing the medulla; and melancholia, by applying the current to the occipital lobes; and has caused auditory delusion to disappear by applying the current to Ferrier's auditory centers in the superior tempero-sphenoidal convolutions.

We have seen similar results follow the use of the galvanic current applied to the head and spine, though always from using a descending current except in tinnitus aurium and other auditory hallucinations. Bright's and Addison's diseases, which, in all probability, are intimately associated with renal ganglia disease in their origin, are greatly benefited by spinal electrizations, and the former has disappeared under its use, if albumen and tube casts are to be taken as indubitable evidence of its existence. Diabetes mellitus, associated with profound melancholia and sexual apathy (loss of sexual desire without spermatorrhœa for six months), we have seen cured by it, conjoined with codia, cannabis indica and neurotic tonics and reconstructives. The miracles of medicine already wrought and still capable of being performed by the aid of galvanism wisely employed as auxilliary to a judiciously prescribed internal therapy, can not yet be exactly estimated, but if we judge even from the known curative verifications of the medicinal power of this agent, our prophetic record must be a liberal and exalted one. It will avoid lengthening this paper, which is intended to be but a brief note on one part of this interesting subject, if we refer the reader to Dr. Althaus' two interesting papers, in Nos. XII. and XIII. of "BRAIN," "*On Some Points in the Diognosis and Treatment of Brain Disease.*"

The cases of cerebral trouble which we now detail may serve to illustrate the one aspect of our subject, which we started out to show:

N. J. W——— is a young unmarried man, of diffident mien, florid complexion ; moderately good flesh ; sleepless ; pulse full and 84 per minute. He is troubled with morbid fears of various kinds, timid, forgetful, and unable to attend to business. His appetite is ravenous, and he is suspicious of the good intentions of his best friends, irritable and cross with them. He is of a sanguine, nervous temperament; some of his family have died of consumption; a sister is excessively nervous and his father died of cancer. Insanity with him is an impending possibility. Cephalic electrization through February and March (1879) and some general treatment in April restored him. He now (1882) attends regularly to business, having only occasional slight recurrences of the head symptoms, which a few days' treatment promptly dissipates.

A young clerk, F. G. W., æt. twenty-three, of full habit, red in the face, with bounding accelerated pulse and constipated ; complains of a severe pressure in the head. Filling a position beneath his aspirations and esteemed by him a menial one, he has become sleepless and melancholy, brooding over what he considers the tyranny of his employer, and lamenting his inexorable adverse fate; he proposes to end his troubles by jumping off the river bridge. A consciousness, however, that something is wrong with his head, leads him to cousult his physician, the distinguished Prof. H, who refers him to me. Coming directly to our office, and receiving a five minutes' electrization, he feels more comfortable, and for the present gives up his purpose of suicide. Given a drachm dose of bromide of potassium in a glass of water and retained in the office half an hour, he is then allowed to go home, with another drachm dose combined with half as much chloral, to be taken as he retires. In the morning he takes a citrate of magnesia and mercurial cathartic, and comes to the office for another seance, which, repeated morning and evening for a fortnight, with bromide and chloral for a few nights, to prolong the tranquilizing effect of the electricity, and later, if he should awaken between midnight and morning, an uncombined dose of chloral, to sufficiently prolong his sleep, and this patient's cure is practically complete. An injunction to take a dose of the bromide mixture at night when inclined to be sleepless, or during the day, if head feels full, and a laxative pill for use when bowels are not free, are all of the precautionary measures prescribed. The patient has had no return of former symptoms at this time (January 1st, 1883).

Mrs. G., æt. thirty-three, married, has borne one child ; has intra-cranial, vascular tension, auditory and visual hallucinations, highly vascular sclerotics and protruding eyeballs. One of the corneæ is scarred from former ulceration. Has had iritis and been under the care of different oculists for inflammatory and exudative conditions of the cornea and anterior chambers of the eye, and it has been pronounced amaurotic and glaucomatous. At the time she came under my care, March 13, 1881, she could neither see objects in her room, or discern light from darkness, though the pupils were dilated with atropine. Her homœopathic oculist informed her that only Providence could save her. An ophthalmoscopic examination revealed no retinal trouble, so that the inference was justifiable that the failure of vision was due to encephalic trouble beyond the ocular fundus (vascular pressure and exudation about the chiasma, the tubercula quadrigemina and augular gyri probably.) The latter condition being especially inferrable from the flashes of light which she has sometimes seen with closed eyes, and the visions of angels which came to her recently during a period of cerebral excitation. Her heart's action was increased in frequency and force, the pulse being 120 when she came under treatment. She had treatment from an irregular electrician and from most of the pathists of this city, without avail. The electrician employed the interrupted current through the head, a procedure not commendable. The patient had marked insomnia, an impaired appetite and sluggish bowels.

Under gelsemium and the bromides and proto-iodide of mercury, with daily cephalic electrization, eight to twelve elements of a constant current battery—descending current—she so greatly improved in the course of a fortnight that she could distinguish all objects in her room, the lineaments of her physician's and husband's faces, the color of her friends' hair and eyes, etc., in short, to see anything but fine print. Her appetite and general condition every way improved, the sclerotics became normally free from blood, and the sanguineous effusion in the anterior chamber began rapidly to disappear. Our visits became less frequent after this—every fourth or fifth day. A minimum dose of hyoscyamia had a very unsatisfactory effect, causing much cerebral excitement, and some kalium iodidum likewise discovered in her an idiocyncrasy, causing, in ten grain doses, an intense diarrhœa. These abortive effects greatly prejudiced the patient against our treatment, notwithstanding we had come in as a *dernier ressort* and greatly benefited her, and during our absence at Richmond, she returned to the infinitesimals.

This patient had formerly suffered from malarial congestions, and some years ago fell down unconscious in an apoplectic fit, from which, in a few weeks, she slowly recovered.

The therapeutic lesson of this case confirms what I have so often before clinically proven, that it has become a fixed article of therapeutic faith with me, that for hyperæmic cerebral states, passive effusions and intra-cranial exudations, constant galvanism is the remedy *par excellence.* The current seemingly acts equally well when applied from above downwards, following the direction of the normal nerve influence, from one hemisphere of the cortex down through the basal ganglia and out at the opposite side of the medulla, as when the electrodes are placed so as to impress the cervical sympathetic, namely, behind carotid at the ramus and angle of the jaw, and at the back of the neck above the seventh cervical vertebra.

Dr. Fdward C. Mann, of New York, in Vol. VII., part 2, of the *London Journal of Physiological Medicine and Mental Pathology*, reports an interesting case of blindness and deafness, resulting from cerebro-spinal meningitis, successfully treated by him with a constant current, in which he details an experience with the electricity quite in accord with our own. We have never, however, cured a case of post meningitic blindness or deafness from this agent, though we have employed it with a view (and we think successfully) of averting this and other horrible sequelæ of this formidable affection.

The following case, however, is much like the preceding. The details of the case appear more at length in a late number of the *Louisville Medical News.* The case was also verbally reported by us along with a number of others, to the Southern Illinois

Medical Society, which lately met at Anna, Ills. The patient is quite well-known in that section of the country :

Rev. L. is a Presbyterian divine residing in Illinois, of intensely studious habits, preparing his weekly sermons with much research and solicitude. The time habitually devoted to this labor is from the middle of the week until the following Sabbath ; his hours of most intense labor being the night time, rarely terminating before midnight on Saturdays, and later, on other nights.

His congregation is influential, critical and appreciative of his work, which he realizes, and while he has labored with solicitude to fill their expectations of him, and he had none of those feelings of depression which come from a consciousness of unappreciated effort, and is not melancholic. He has, however, realized of late the failure of his mental powers for prolonged studious effort, and has become conscious that he must get relief or abandon his calling.

His symptoms, when he first came under observation, were protrusion of the right eye and inability to distinguish light from darkness with it; cephalagia with inability to labor mentally without intensifying it; full pulse, 84 per minute, and increased temperature, 99.5 F. on side of blindness; sluggish bowels; an ill-at-ease sort of feeling in the daytime, and incapacity for sufficiently prolonged, dreamless and refreshing sleep, to daily recuperate him. He had no catarrh, and there were objective noises in his left ear. Otoscopic and ophthalmoscopic examination gave negative results. Æsthesiometric examination gave abnormal and lessened tactile sensibility in the terminal branches of the trifacial. Giddy sensations were complained of, and his appetite was somewhat impaired. The renal, hepatic, enteric and cardiac functions, save the ganglionic excitation in the latter, were not appreciably abnormal.

The condition of this patient was one of partial paralysis of the vaso-constrictor nervous system, due probably to malarial influences as the pre-determining cause, and to psychical overstrain as the immediate exciting cause. I regard the cerebral pathological condition as one of psychically induced cerebral hyperæmia with meningeal hyperæsthesia and cortex irritability.

The treatment consisted mainly in cerebral galvanization with the constant descending current daily, of varying strength, enforced brain rest, and chemical restraint imposed by the sodium and potassic bromides in afterpart of day and night, together with all rational efforts to restore trophic and waste cerebral equilibration. The following further history of this case is given by the patient himself:

"I came into Southern Illinois in the spring of 1876. After being here about a month or two, I took chills and fever. I was troubled with them for about one year. After getting clear of them I began to be troubled with what my physician here called nervous headache. As time passed this grew more troublesome until I had it half or more, probably of my time. In September, 1881, I went north to spend a few days, and while there had severe pains in my head, and was under the necessity of remaining in a dark room for about forty-eight hours. During that time I lost the sight of my right eye entirely. Came back home and staid till last November, when my left eye became somewhat affected, when I placed my case in your hands, or under your treatment. My sight was perfectly restored before I left the city, and since I have had no trouble whatever, so far as they are concerned. I have been able to work ever since I returned home. Have done harder work and more of it than for three or four years before. My head does not trouble me much now. I think I have had headache but once during the last month. I eat well, sleep well, I feel well generally, but I am exceedingly nervous."

The patient has lost thirty seven pounds in weight, and complains that he can hardly hold a paper still enough to read it. He will require further treatment for the general nervous symptoms, but the cerebral hyperæmia, meningeal hyperæsthesia and cortex irritability were subdued by the treatment and the concomitant blindness due to the cerebral condition, disappeared simultaneously.

In our view, while the effect of cephalic electrization is to produce diminished circulation within the brain, this effect is often undoubtedly contributed to by a concomitant or precedent tranquilization of the cerebral cells, whose state of excitation induces hyperæmia. The effect on the brain and its meninges may be primary, on the circulation secondary to, and as a consequence of, the tranquilization of the excited cell movements, in some cases. *An essential property of the constant descending galvanic current in induced cerebral hyperœmia is that of a tranquilizer of irritable nerve tissue*, secondarily contributing to the contraction of over-distended vessels. It acts on the irritable brain like bromides, hyoscyamin and chloral, vasomotor results being secondarily induced when there is over vascular distension as well as primarily accomplished.

Certain effects of cephalic electrization are too immediate to be the result solely of the circulatory changes made by it. For example: the prompt relief of migrain and other hyperæsthetic neuroses of the meninges, as well as in all forms of anæmic and congestive cephalalgias; though it is undoubtedly more effective in the latter.

It is a well-known fact, in regard to certain hypnotics, that they first accelerate and augment in force the cerebral circulation, even while the obtunding of consciousness and the gradual quiescence of the brain is being accomplished, so that to attribute their sleep-inducing power to their influence over the vasomotor system is not logical. They induce sleep under varying states of the circulation, as in opium, alcohol, chloral and bromide slumber, the state of the circulation being different in all. We may fall into error if we attribute the effects of electricity solely to its vasomotor influence.

